# Editorial: Community series in recent advances in potential biomarkers for rheumatic diseases and in cell-based therapies in the management of inflammatory rheumatic diseases, volume II

**DOI:** 10.3389/fimmu.2024.1530739

**Published:** 2025-01-03

**Authors:** Katarzyna Bogunia-Kubik, Philippe Saas, Eric Toussirot

**Affiliations:** ^1^ Hirszfeld Institute of Immunology and Experimental Therapy, Polish Academy of Sciences, Wroclaw, Poland; ^2^ Institute for Advanced Biosciences, Team: Epigenetics, Immunity, Metabolism, Cell Signaling & Cancer, Inserm U 1209, CNRS UMR 5309, Université Grenoble Alpes, Grenoble, France; ^3^ Etablissement Français du Sang Auvergne-Rhône-Alpes, R&D Laboratory, Grenoble, France; ^4^ Université de Franche Comté, INSERM Centre Investigation Clinique CIC-1431, CHU de Besançon, Besançon, France

**Keywords:** TNF inhibitors, biomarkers, cell-based therapy, inflammatory rheumatic diseases, psoriatic arthritis, rheumatoid arthritis, spondyloarthritis, systemic lupus erythematosus

This Research Topic focuses on recent advances in the identification of diagnostic, prognostic and predictive biomarkers for inflammatory rheumatic diseases (IRDs), as well as recent advances in cell-based therapies used in their treatment (1). The second volume is composed of 3 review articles and 7 original articles, and the manuscripts collected are especially dedicated to biomarkers in IRDs. They present the effects of various approaches leading to the identification and description of biomarkers, such as: (i) a review of existing biomarker recommendations and meta-analysis of existing data (e.g. of epigenetic and DNA methylation studies); (ii) application of more laboratory-based analyses, such as measurement of single protein concentrations, circulating exosomal miRNA and serum proteome profiling, detection of specific antibodies in serum and saliva; and (iii) implementation of newer biostatistical methods including machine learning. Consequently, in addition to well-known associations, several new findings in the field of IRD biomarkers are provided.

Liu et al. reviewed and formulated guidelines for biomarkers in the diagnosis and assessment of patients with axial spondyloarthritis (axSpA). They highlighted the usefulness of currently applied HLA-B27 testing in patients with suspected axSpA and regular C-reactive protein/erythrocyte sedimentation rate monitoring as specifically recommended for axSpA evaluation (2).

Mangoni and Zinellu in their systematic review and meta-analysis focused on the pathophysiological role of neopterin known as a biomarker of inflammation and oxidative stress, suggesting that it may be useful in identifying rheumatic diseases. Neopterin is an organic compound belonging to the pteridine class of heterocyclic compounds and belongs to the chemical group known as pteridines. It is synthesized by human macrophages after interferon-gamma stimulation and serves as a marker of activation of the cellular immune system.

The role of epigenetic regulation of gene expression and function of DNA methylation was discussed by Wang et al. who argue that genome methylation analysis may be a beneficial tool to aid in the early diagnosis of Sjögren’s syndrome (SjS). Interestingly, in addition to the human leukocyte antigen (HLA) locus, several regions were found to be differentially methylated in SjS patients, including genes regulated by type I interferon, the runt-related transcription factor gene (RUNX1), lymphotoxin-α (LTA) and myxovirus A resistance protein (MxA).

More experimental analyses, such the application of molecular profiling and gene expression studies for biomarker detection was also proposed. Angioni et al. performed a transcriptomic analysis of whole blood and a comparison of molecular profiles between psoriatic arthritis (PsA) patients in clinical remission after TNF inhibitor (TNFi) treatment, PsA patients with active disease and healthy controls. The results pointed the role of dysregulation of two genes involved in the processes of inflammation perpetuation and bone metabolism. Downregulation and upregulation of FOS and CCDC50, respectively, were identified as contributing to the pathophysiology of PsA as described in a study on clinical remission in PsA patients treated with TNFi.

Serum proteome profiling was used in another study by Cuesta-López et al. investigating age- and sex-matched control patients with newly diagnosed rheumatoid arthritis (RA) patients, and patients with established RA (with a disease duration of more than 25 years). An additional longitudinal study was conducted on two cohorts of RA patients treated with methotrexate or tofacitinib for 6 months. By analyzing their cardiovascular and cardiometabolic proteome (examining serum profiling of 184 proteins using the Olink technology platform), the authors were able to identify changes in serum proteins associated with cardiovascular disease in RA patients and identify candidate protein biomarkers to distinguish RA patients from healthy individuals (such as elevated levels of CTSL1, SORT1, SAA4, TNFRSF10A, ST6GAL1 and CCL18). They were also able to show how methotrexate and tofacitinib affect serum levels of these proteins, and to identify SAA4 as a potential biomarker of response to these therapies.

Serum and salivary antibody levels against *Porphyromonas gingivalis* (*P. gingivalis*, a major periodontal pathogen) were investigated by Svärd et al. in a Swedish RA patient cohort. The results indicated the local production of IgA directed against *P. gingivalis-*specific Arg-specific gingipain B (anti-RgpB) in the salivary glands, which is not accompanied by systemic antibody production. Higher levels of IgA anti-RgpB antibodies in saliva were detected in RA patients compared to healthy controls. However, despite some common features and a potential link between RA and periodontitis (e.g., elevated levels of anti-citrullinated peptide antibodies [ACPAs]) (3), anti-RgpB antibodies were not found to be associated with RA disease activity, periodontitis or serum IgG ACPAs.

Four remaining original articles addressed biomarkers related to lupus nephritis (LN) development, the disease onset, activity, and response to treatment.

The study by Li et al. aimed to assess whether serum levels of human epididymis protein 4 (HE4) can identify pathological classes of LN in adults and children with systemic lupus erythematosus (SLE). The study was conducted on three cohorts of SLE patients (those without LN as well as LN patients with adult or childhood onset) and showed an association of higher serum HE4 levels with adult onset LN. It was also observed that among patients with adult onset LN, elevated HE4 levels were more common in patients with proliferative LN and in patients with chronic class IV lesions.

It was also found that urinary L-selectin (uL-selectin) level may act as a novel biomarker of disease activity and renal histopathology in LN. Moreover, it may reflect treatment response in LN patients during follow-up, as uL-selectin concentrations decreased significantly in the complete renal remission group. This was reported by Shen et al. in a study investigating two independent cohorts, a Chinese cohort and a US cohort of SLE patients and controls.

Chen et al. analyzed and compared circulating exosomal microRNA molecules in the serum of SLE patients with or without LN. They detected significantly higher levels of exosomal hsa-miR-4796-5p and hsa-miR-7974 in LN cases compared to SLE patients without LN. The levels of these miRNAs positively correlated with proteinuria and SLE disease activity index (SLEDAI), and were significantly elevated in patients with LN compared with other autoimmune nephritis conditions such as immunoglobulin A nephropathy (IgAN) and diabetic nephropathy (DN).

Therefore, it appears that laboratory analysis of uL-selectin levels, serum He4 levels and profiling of exosomal miRNAs in patients’ sera may help predict the onset and course of LN. However, the use of statistical tools such as machine learning may also be helpful in a more complex analysis, as shown by Yang et al., offering a reliable non-invasive diagnostic tool for SLE patients when renal biopsy is not possible or safe.

Yang et al. performed a retrospective analysis of clinical and laboratory data from patients diagnosed with SLE and renal involvement who underwent renal biopsy aiming to develop and validate machine learning models to predict the occurrence of proliferative lupus nephritis (PLN). Their study confirmed the efficacy of traditional indicators such as anti-double stranded DNA (dsDNA) antibodies, complement levels, serum creatinine and urinary red and white blood cells in predicting and differentiating PLN, and also demonstrated the potential value of previously controversial or underutilized indicators such as serum chloride, neutrophil percentage, serum cystatin C, hematocrit, urine pH, routine red blood cells and immunoglobulin M in predicting PLN.

In conclusion, employment of various experimental approaches, laboratory and statistical tools provides a comprehensive perspective on the inclusion of a wider range of biomarkers in the diagnosis, prediction and treatment outcome of IRDs.

As Volume III of our Research Topic has been initiated, we invite authors to submit their manuscripts.
